# An effective internet-based system for surveillance and elimination of triatomine insects: AlertaChirimacha

**DOI:** 10.1371/journal.pntd.0011694

**Published:** 2023-10-16

**Authors:** Laura D. Tamayo, Carlos E. Condori-Pino, Zoee Sanchez, Raquel Gonçalves, Fernando S. Málaga Chávez, Ricardo Castillo-Neyra, Michael Z. Levy, Valerie A. Paz-Soldan

**Affiliations:** 1 Zoonotic Disease Research Laboratory, One Health Unit, School of Public Health and Administration, Universidad Peruana Cayetano Heredia, Arequipa, Perú; 2 Department of Tropical Medicine and Infectious Disease, Tulane University, School of Public Health and Tropical Medicine, New Orleans, Lousiana, United States of America; 3 Gerencia Regional de Salud, Arequipa, Perú; 4 Department of Biostatistics, Epidemiology and Informatics, University of Pennsylvania, Philadelphia, Pennsylvania, United States of America; ICIPE: International Centre for Insect Physiology and Ecology, KENYA

## Abstract

Vector-borne diseases remain a significant public health threat in many regions of the world. Traditional vector surveillance and control methods have relied on active and passive surveillance programs, which are often costly and time-consuming. New internet-based vector surveillance systems have shown promise in removing some of the cost and labor burden from health authorities. We developed and evaluated the effectiveness of a new internet-based surveillance system, “AlertaChirimacha”, for detecting *Triatoma infestans* (known locally by its Quechua name, *Chirimacha*), the Chagas disease vector, in the city of Arequipa, Peru. In the first 26 months post-implementation, AlertaChirimacha received 206 reports of residents suspecting or fearing triatomines in their homes or neighborhoods, of which we confirmed, through pictures or inspections, 11 (5.3%) to be *Triatoma infestans*. After microscopic examination, none of the specimens collected were infected with *Trypanosoma cruzi*. AlertaChirimacha received 57% more confirmed reports than the traditional surveillance system and detected 10% more infested houses than active and passive surveillance approaches combined. Through in-depth interviews we evaluate the reach, bilateral engagement, and response promptness and efficiency of AlertaChirimacha. Our study highlights the potential of internet-based vector surveillance systems, such as AlertaChirimacha, to improve vector surveillance and control efforts in resource-limited settings. This approach could decrease the cost and time horizon for the elimination of vector-mediated Chagas disease in the region.

## Author summary

Insects that spread diseases are still a problem in many parts of the world. Monitoring and controlling them can be expensive and time-consuming, but new internet-based methods have shown promise in reducing workload and costs. We developed AlertaChirimacha, an internet-based surveillance system to detect *Triatoma infestans*, a species of insect that spreads Chagas disease. AlertaChirimacha was tested in Arequipa, Peru, and in the 26 months after it was implemented, it received 206 reports from residents who suspected they had these insects in their homes, 11 were confirmed by our team. AlertaChirimacha detected more infested homes than the traditional methods combined. AlertaChirimacha was also effective in reaching people, getting them to engage, and respond to reports. This approach can improve efforts to control insect-borne diseases in places with limited resources and make it less expensive to eliminate vector-mediated Chagas disease.

## Introduction

Vector surveillance and control have been the main, and, in some cases, the only approach to reduce the morbidity and mortality caused by vector-borne diseases [1]. Vector surveillance programs have traditionally relied on two components: active surveillance requiring physical searches led by health personnel, and passive surveillance relying on affected individuals’ reports. Active surveillance is costly and time-consuming, but generally can provide accurate information for the early detection and prevention of outbreaks [2]. Passive surveillance is less expensive (for the health authorities) and can provide useful data but requires an efficient system for information uptake and rapid response. The two approaches together often are greater than the sum of their parts [3].

The Chagas disease vector, *Triatoma infestans*, has been controlled via indoor residual insecticide application across multiple regions of southern South America [4] through a coordinated program known as the Southern Cone Initiative (INCOSUR) [5–7]. Continued entomological surveillance following these successes, combined with an extensive and rapid control, is essential to avoid the re-emergence of the insect. The city of Arequipa posed one of the greatest challenges to the Southern Cone Initiative as the vector had spread widely through the dense urban environment [8,9], but great progress has been achieved during the last years in reducing household infestation. However, the closer control programs approach elimination of vector-borne transmission of *Trypanosoma cruzi*, the more difficult it becomes to maintain effective surveillance–as it becomes less of a priority health concern, personnel and financial resources are allocated to other programs.

The adage of “looking for a needle in a haystack” is appropriate: fewer vectors are harder to find, resulting in a decrease in perceived risk at the community level, dwindling motivation among vector control specialists and health professionals to search for the vector, and reduced attention and allocated resources/budget by a health system with multiple other priorities [10–13]. New internet-based vector surveillance systems hold great potential to strengthen surveillance and remove some cost and labor burden from health authorities [14,15]. These have been designed and tested in different countries, with varying success. Some programs have seen success in community engagement for vector reporting via apps or internet-based systems [16–21]. Several programs have also described the importance of community engagement in the vector-control process–a process that creates and builds relationships and allows for information exchange between public health professionals and members of the community [19,22,23]. This bilateral engagement aids in the collection of data and identification of vector foci by promoting increased education and awareness for community members [18,23]. Timely and professional responses to questions or messages from the community about possible infestations are key to showing that the community’s participation is valued and that vector surveillance and control requires everyone working as part of a larger system [13], as well as tools for reducing barriers in communication and reporting. Rapid responses to community members keep them engaged in the process and encourage further action. Ultimately, these programs have been described to have large reach into the population, and reduced costs to the health system [16].

In response to the above-mentioned challenges to the triatomine surveillance system, combined with the lockdowns due to Covid, we developed an internet-based triatomine surveillance and response system for the city of Arequipa: “AlertaChirimacha” for detecting *Triatoma infestans*, a triatomine insect known locally by its Quechua name, *Chirimacha*). Moving away from a traditional hierarchical vector surveillance and control system, AlertaChirimacha is designed to facilitate a streamlined integrated vector surveillance and control program [24].

## Methods

### Ethics statement

The research protocol was approved by the ethical review committees from Universidad Peruana Cayetano Heredia (Approval number: 103096), and University of Pennsylvania (approval identification number: 833122). Only entomological data were collected.

### Study site

AlertaChirimacha was launched in Arequipa, an Andean city of ~1 million inhabitants located 2,300m above sea level in southern Peru [25]. A triatomine control campaign (“campaign” from hereon) conducted between 2003 and 2018 has nearly succeeded in eliminating *Triatoma infestans* from affected districts. Following the campaign, a conventional surveillance system was put in place in a rolling manner following insecticide application in each district [26]. The conventional system includes both passive and active surveillance. The passive system requires residents who suspect they have triatomines in their home to capture an insect and bring it to a health post or center for identification. If the insect is confirmed as *Triatoma infestans*, the regional health authorities or our staff conduct an inspection of the house that reported the insect and adjacent houses, expanding outward until two successive negative houses are found. Infested houses and their immediate neighbors are treated with insecticide, and residents in the area are educated and made aware of the need to be vigilant for this insect. AlertaChirimacha was first launched in October of 2020; here we report results of the first 26 months (until December 2022).

### AlertaChirimacha

AlertaChirimacha was designed to work through three main mechanisms: 1) extensive and inexpensive **reach** (coverage) into people’s homes to communicate educational messages, 2) **bilateral engagement** between health authorities, our research team, and the community to allow for questions and answers, as well as diffusion of targeted information, and 3) a **rapid and efficient response** to community based vector reporting.

**Fig 1:**
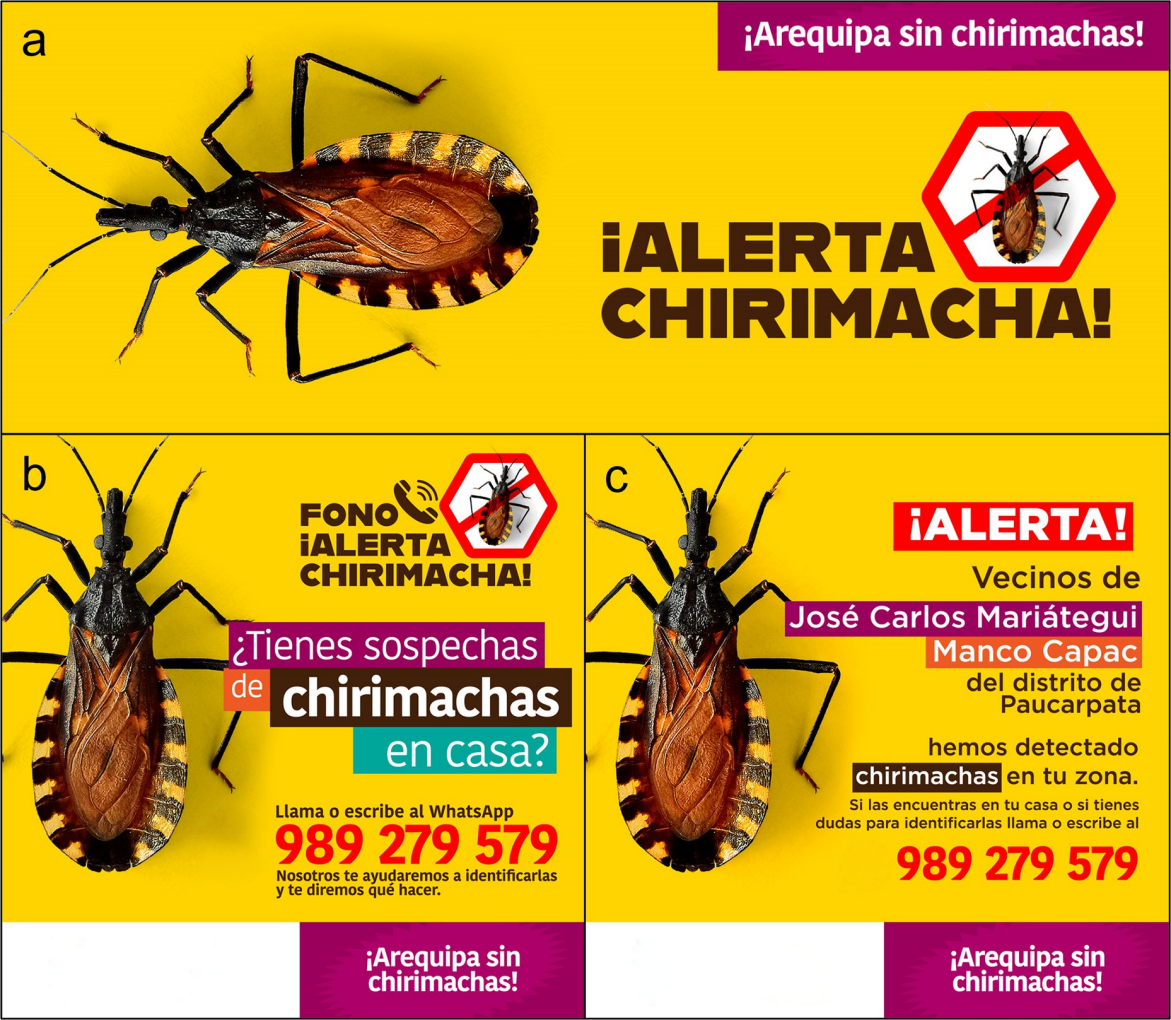
AlertaChirimacha Facebook page and posts. **a**. Image of home page of AlertaChirimacha on Facebook (https://www.facebook.com/AlertaChirimacha). **b**. Design of first post, promoted through Facebook and flyers, that aims to inform the community of the new system and the number to contact. **c**. Design of a target post informing the population that *chirimachas* (local name for triatomines) have been found in their neighborhood and asking them to look for and report any if found at home.

**Reach:** Through formative pilot work, we determined that Facebook and WhatsApp are widely used in our population, and designed an educational video (S1 video). The video has two goals: 1) clarify the reporting processes the population can use, and 2) educate the population on what *T*. *infestans* looks like (or its dry fecal spots in a home), where it is most likely to be found, as well as information about Chagas disease. We paid for posts–at about 50 USD per ad–that were released every 3 months throughout the region of Arequipa. The Facebook Page was linked to the two Facebook pages of the Gerencia Regional de Salud y Red de Salud Arequipa-Caylloma (equivalent to Arequipa’s Regional Ministry of Health and Regional Health System) with 174,000 and 11,000 followers respectively (Fig 1).

To ensure widespread reach among those who do not use Facebook (i.e., elderly), we also promoted use of AlertaChirimacha WhatsApp for reporting suspected triatomines using flyers and posters: some were posted at health facilities, some in *bodegas* (small community stores), some distributed door to door by vector control specialist during active surveillance work and in neighborhoods within 200 meters of areas reporting infestation.

**Bilateral engagement:** Community engagement was key to AlertaChirimacha, and for this, it was important to create a system that allowed for bilateral communication; ultimately, this would generate a dynamic system that community members could go to and resolve questions quickly, as well as generate trust in the expertise of those addressing the questions thoroughly. Hence, a biologist checked, and responded thoroughly to, all comments on the AlertaChirimacha site within 2–3 days. On all diffused communications (ads, flyers and posters), we also promoted a phone number that people could use directly as well to call or message us: to send pictures, to ask more questions, to report suspected insects. Residents could send pictures of the insect or signs of its presence; if residents were unable to provide a picture, they could describe what they saw. For every picture received, the biologist confirmed the species and responded to the sender. In the case of *T*. *infestans*, a vector control specialist was sent to conduct a thorough inspection of the household and its immediate neighbors as described above. In the case of a non-target insect, the biologist responded by identifying the insect species, providing reassurance that it was not dangerous, and also explaining, usually with images, how the reported insect differs from *T*. *infestans*. Moreover, if the photo was identified as a bed bug (*Cimex* species), the biologist sent an informational pamphlet (Spanish version of the guide "Preventing and Getting Rid of Bed Bugs Safely" from the Department of Health and Mental Hygiene of New York) [27], and answered related questions.

For reports without a photograph, the biologist asked more questions related to the characteristics of the insect, including the place and time of day where it was found. In some cases, the description was enough to rule out triatomines—such as for insects found among garden plants (extremely unlikely for *T*. *infestans*). The individual was told what the insect could be and was asked to try to take a picture of the insect if they encountered it again. However, if there was any doubt, a vector control specialist was sent to inspect the house.

**Rapid and efficient response:** The conventional passive reporting system contains multiple points at which critical information can be lost. There is limited quality control within the system to ensure that residents who manage to catch an insect in a bag and bring it to a health post, receive follow up inspections and insecticide treatment when warranted. Currently, it is impossible to calculate the true number of triatomines that were never reported or reported and never registered by the health system. Hence, following triatomine confirmation, either by photo or inspection, our research team, in coordination with Arequipa’s Regional Ministry of Health, sent a vector control team to the reporting house within 7 days to perform inspections. Households where at least one Chagas disease insect vector was identified, regardless of its infection status with *Trypanosoma cruz*i, were treated with residual insecticide following Peruvian Ministry of Health established protocols. All specimens, excluding first instar nymphs, underwent examination for the presence of *Trypanosoma cruzi*. For this purpose, each insect was gently compressed abdominally to extract fecal droplets. These droplets were then diluted with a drop of 0.9% saline solution and placed under a 22x22 microscope slide. Subsequently, the sample was observed under 400x magnification to detect active trypanosomes [28]. Additionally, within the context of a larger cluster randomized trial [24], when a triatomine was detected in parts of the city assigned to the intervention area, we designated an area of 200 m around the infested household for increased activation of the surveillance system. Within this intervention zone, we: 1) visited all households, conducted inspections, and distributed flyers advertising AlertaChirimacha, 2) put up posters in all local shops willing to let us do so, and 3) created and advertise Facebook posts for 10 days for residents living within 1 km of the infested house (smallest area that could be created for these posts on Facebook, and this range changed over time due to FB regulations). This intervention served to inform that an infestation was found in the area and to encourage residents in the area to inspect their homes for the triatomine and report any findings of a suspected bug or its dry fecal spots via WhatsApp or phone call (Fig 1C).

If we found additional infested houses near the original focus, a follow-up post was released at days 10 and 30 after the initial post. Subsequent posts informed neighbors that more houses have been found positive and asked them to stay alert to the presence of triatomines and report them if found. It is impossible to keep a community on high alert indefinitely, hence, once we did not find additional infested houses, we posted a message 30 days after the last publication, explaining that the affected houses had been treated with insecticides and the infestation was controlled.

### Analysis

Various metrics were used to assess the success of the three mechanisms. To assess the reach of AlertaChirimacha, we used the Facebook metrics to evaluate “reach” (number of people who saw the ad at least once) and total number of reactions, comments, and times the posts were shared and clicked on) for each published ad. We also assessed reach via the number of posters still visible in local stores 90 d after being posed. Additionally, based on data collected at the time of insect reporting to our team via phone calls or Whatsapp, we present the number of houses that received an AlertaChirimacha flier. Regarding bilateral engagement, all questions and comments on Facebook were documented in an excel database, as well as the response given to each question or comment by our biologist. We reviewed all questions and comments, and inductively coded all responses to 4 main themes: 1) Questions or information about the vector, the disease, or the reporting system, 2) Reports about the vector, 3) Expressions of fear, concern or appreciation regarding information provided, and 4) Other random topics. Findings are presented in a graph, along with quotes depicting the types of comments of the three main categories. Regarding rapid and efficient response, we describe the average response time between the receipt of a positive complaint, followed by an inspection and treatment.

## Results

**Fig 2:**
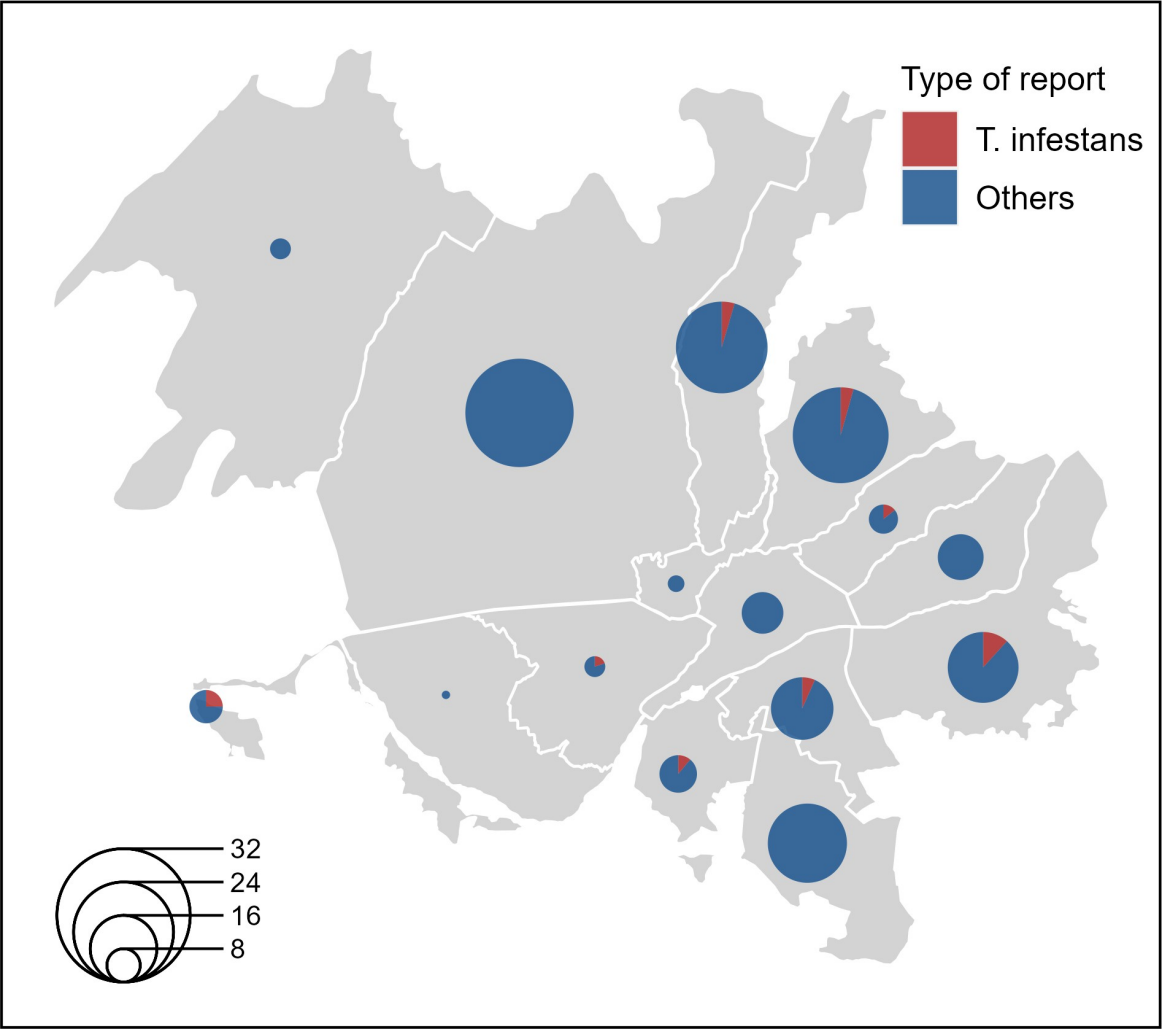
Distribution of AlertaChirimacha reports by district in the metropolitan area of Arequipa, Peru. The pies represent the total number of reports; sections in red represent reports confirmed as *Triatoma infestans*.

All results are based on findings in the first 26 months post implementation of AlertaChirimacha. We received 206 reports of residents suspecting or fearing triatomines in their home or neighborhood in Arequipa. Most reports were for non-target insects (Fig 2), such as a phytophagous insect of the genus *Vazquezitocoris* (23%). Eleven (5.3%) reports were confirmed to be *Triatoma infestans* in Arequipa. We also received 24 reports from 15 provinces outside of Arequipa, three of which were confirmed as Chagas vectors (two were *Panstrongylus chinai* and one a member of the *Triatoma* genus which could not be fully identified from the report photograph (see S1 Table). We receive 19 additional reports that did not report location. Notably, more infested households were detected and confirmed via AlertaChirimacha (11) than through traditional surveillance reports during the same period: passive surveillance (7), and active vector control specialist-led surveillance (3, out of 9,574 inspections by trained personnel during that time period). None of the examined insects were infected with *Trypanosoma cruzi*. Regrettably, vector insects linked to reports from provinces outside Arequipa could not undergo testing due to logistical complications to transport samples to our laboratory in Arequipa.

**Fig 3:**
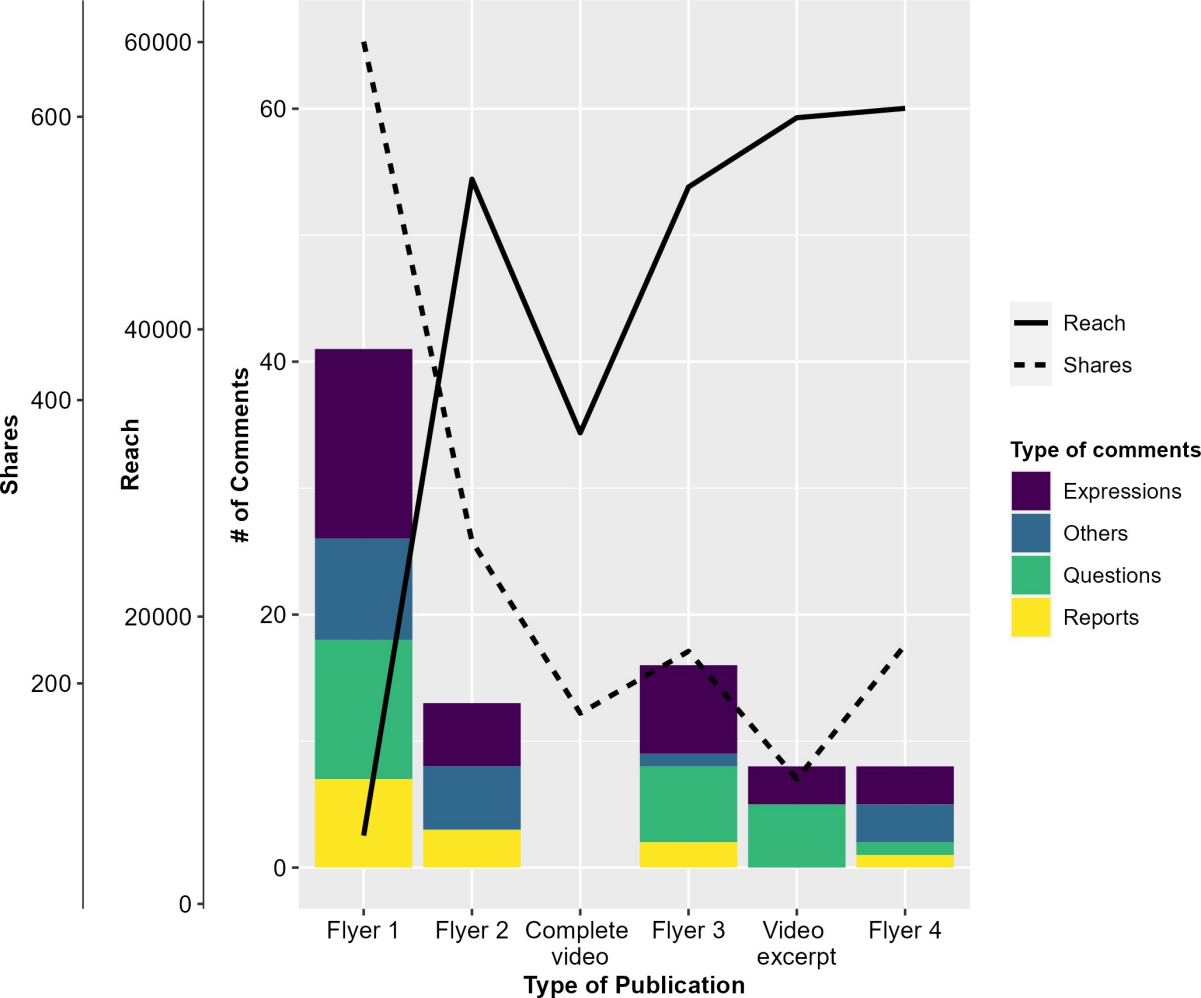
Facebook metricts and comments for AlertaChirimacha posts. The reach (number of individuals exposed to the post), shares (number of individuals who shared the post) and comments (categorized as expressions, questions, reports and other) for AlertaChirimacha posts between October 2020 through December 2022.

**Reach:** In 26 months, we posted 32 Facebook ads: 6 were general posts about Chagas disease and its vector. In addition, a total of 4,313 households were informed about AlertaChirimacha through door-to-door communication campaigns. Our initial Facebook post, a simple flyer introducing AlertaChirimacha (Fig 1), had a relatively small reach (2,528), but was the most shared post among Facebook users (653). In subsequent posts, the reach increased significantly, peaking at 60,028 for the final post. However, as the number of posts increased, the number of shares decreased (Fig 3). On the other hand, the video publications had a strong reach (34,368 and 59,287) but did not generate much interaction. There was no trend in reactions or sharing of targeted posts over time (see S2 Table).

In the 200-meter radius around the infestations, 395 out of 400 stores agreed to have a poster pasted on the wall. Those who refused indicated that they did not have "space" on their wall or did not want posters on their wall. When the stores were revisited 90 days after completion of the work, approximately 85% of these posters were still visible to the population; the other 15% were covered by product advertisements or had simply been removed. This information diffusion and inspections within the 200 m radius, combined with the targeted posts on Facebook, allowed us to detect 5 additional infested houses in addition to those that would have been detected if only the traditional protocol of the Regional Ministry of Health was followed (to inspect adjacent houses until vector control specialists reach two consecutive negative households).

**Bilateral engagement:** Most comments on the posts were questions regarding the vector or the disease, such as why the triatomine is dangerous, where it lives, and how to prevent it from getting into homes.

Other frequent comments on AlertaChirimacha were from people adding to the information about triatomines and Chagas disease, occasionally describing their past experiences with the vectors or the illness. For example, some comments came from Venezuelan migrants who recognized the insects and indicated the common name for similar Chagas disease vectors in their country.

“*Chagas disease is the disease*, *but its bite [triatomine] does not produce it*. *What produces it [transmission] are its feces and urine*. *In case of a bite*, *do not rub it because it would result in a greater risk of infecting yourself by scratching the urine or excrement into the wound*. *Another important fact in case of a bite is not to kill the insect; on the contrary*, *you must catch it alive and thus be able to take it to analyze it to know if it is infected or not; this is for your peace of mind*, *since the disease is positive years later from its contagion*.”

“*In my country it [triatomine] is called chipo and causes Chagas disease which is an inflammation of the heart but it occurs after 50 years of age*. *If you see it*, *do not kill it because the liquid that it releases is harmful*.”

“*These bugs have wings*, *are horrible smelling*, *dark in color*, *and are a little long*. *They hide in dark places*, *under chairs*.*”*

Additionally, there were expressions—both in text and through emojis—of alarm, fear and concern, as well as of appreciation for the site. Multiple people tagged their friends to spread the message. A few people used the comments to report that they had seen the insect or suggest areas where these might be found; however, these reports about “others” generally were sites recognized to be cluttered, dirty, or with animals.

“*My neighbor lives like in a garbage dump*. *He has no retaining wall and there he throws his garbage*. *He says he has a lot of money*, *but he doesn’t make his retaining wall… I am sure he also has triatomines*. *Who can I contact*?”

There were also limited comments spreading false information, such as saying the insects are harmless or even that they ’don’t exist’. We addressed these on a case by case basis, eliminating a few comments that were political or offensive, and ignoring others. We note that these counterproductive comments did not gain traction and received few or no likes or forwards.

**Rapid and efficient response:** A total of 68 homes have been treated (20 infested and 48 considered at risk due to their proximity to infested houses) in response to the detection of infested homes through AlertaChirimacha. We were able to respond within 1–3 days to all insect reports via AlertaChirimacha. In the case of positive reports, all households were inspected within one week of the report, and all infested households were treated immediately following confirmation (though we experienced some delays due to rains, the pandemic, and political protests). The response rate and time through the traditional reporting system can not be estimated since many reports are lost in the process, and the dates of reports are not always noted. On a few occasions individuals who reported triatomines via AlertaChirimacha noted that they had reported the insect before through the traditional system and had received no response, in some cases even six months later; hence, their decision to contact this number.

## Discussion

AlertaChirimacha, born out of necessity during the pandemic lockdowns in Peru, has proven a critical tool for surveillance and control of urban Chagas disease vectors. Over the course of two years, more infested households were detected and treated via AlertaChirimacha than the two traditional surveillance methods–active search by vector control specialists and passive receipt of insects at health posts–combined. The tool also has great potential for sustainability due to its low human resource demand, and, if the rate of detection of infested houses continues to surpass existing methods, could decrease the cost and time horizon for the elimination of vector-mediated Chagas disease in the region.

In the epidemiological context of Arequipa where the elimination of the sole vector of Chagas disease is near, the foci identified over the course of 26 months is not negligible. Barbu et al estimated a residual infestation prevalence following insecticide application in treated districts of 1.2% [26]; over successive years, residual and re-emerging foci have been identified and eliminated, resulting in a further reduction in prevalence. While AlertaChirimacha is not designed for estimates of the infestation prevalence in the area and cannot be used to assess the disruption of transmission, it can aid in the ongoing efforts to eliminate the insect from the city.

The objective of AlertaChirimacha was to improve surveillance by engaging the community to report triatomines using a streamlined communication approach that was feasible from home and during a lockdown. There were many reported challenges to the traditional surveillance and control system: residents were required to capture a dangerous insect in a bag and take it to a health facility, and once there, residents were expected to navigate the facility hierarchy to make sure the insect reached the right hands (e.g., vector control specialists). Even if it did, there was no assurance that the report would make it to the vector control authorities of the Regional Ministry of Health which oversees the vector control specialist and allocates insecticide. AlertaChirimacha, instead, created a direct line of communication between residents and the vector control authorities of the GERESA (Regional Ministry of Health). It also integrated reporting, confirmatory inspections, and treatment; hence, eliminating gaps in the flow of information and facilitating prompt and efficient responses.

AlertaChirimacha required a behavior change from the population: moving from taking suspected insects to their health post, to taking a picture of the insect and sending it via WhatsApp or Facebook Messenger. The Behavior Change Wheel is a theoretical model that describes that behavior change requires interventions that focus on building capability, provide opportunity for change, and increase motivation (COM-B)–all of which were important components of AlertaChirimacha [29]. Via Facebook messaging and videos, we built community capability for identifying the triatomine and knowing where to look for it, we provided feasible opportunities for streamlined reporting via WhatsApp on their phones and Facebook Messenger, and by ensuring a quick and timely response we increased motivation for reporting. Moreover, AlertaChirimacha was designed using fundamental principles of community health education and promotion, such as community engagement and prioritizing community concerns (by responding to and providing information about all insect pictures that were worrisome to them) [30]. Other internet based vector reporting programs have been implemented for ticks, mosquitos, and triatomine insects [16–19,21,31], and, in contrast to AlertaChirimacha, many have faced challenges for sustained community use or data integration [21].

The direct line of communication with the research team and the health authorities in charge of overseeing the application of insecticide did not create an undue burden on the control program. The number of reports received was large, but these were easily and quickly answered (by LDTQ or CEC). Everyone who reported any type of insect received an answer to their concern, whether it involved a triatomine or not. A positive feedback loop was thereby created, where individuals learned that reports were taken seriously and responded to quickly. We suspect that this, more than anything, led to sustained use of the system [13]. The ability to share pictures of insects also increased efficiency—for the community and for the system. We were able to assuage fears and close out all queries via text, avoiding hundreds of unnecessary inspections. Prompt and structured responses were not only provided to those who submitted reports, they were also received by those who made queries and comments on Facebook posts. This not only made users feel that their participation was valued but also helped other Facebook users to trust the system.

Our reporting system and our study carry limitations. While access to smartphones and Facebook are extremely high in Peru [32,33], it is likely that the elderly who may not be as connected to social media and that the most marginalized populations who may have the least digital access were less informed. The inclusion of old-fashioned physical pamphleting and the placement of posters in bodegas of affected areas may have contributed to the overall success of the system. Another limitation is the use of Facebook reaction metrics (i.e., likes, shares) to evaluate the “reach” component of AlertaChirimacha, as they may represent populations outside of the study area or regions where the presence of other non-triatomine insects may trigger responses. Likewise, the sensitivity of microscopy for the detection of *T*. *cruz* is imperfect and it is possible that there were some infected insects that we missed. Nevertheless, it is noteworthy that we have not identified the presence of the parasite in urban Arequipa since 2015. It is also worth noting that the nature of the reported data does not lend itself to making precise estimates regarding the prevalence of infestation.

There are many policy and programmatic implications of this work. Although a detailed cost-effectiveness study is necessary to compare resources spent on AlertaChirimacha compared to the traditional surveillance system, AlertaChirimacha requires limited personnel resources for reaching and engaging with households and ensuring a timely response to reported cases–it is likely much more cost effective. It uses technologies that were not available twenty years ago that allow us to “enter” tens of thousands of households. In fact, it even reached households outside of the city of Arequipa, providing evidence that this strategy could cover a much larger territory and could potentially be used for other vector-borne or zoonotic diseases that require community reporting, such as that for ticks and canine rabies. Only time will tell us if this strategy maintains momentum: our initial 26 months have shown that the positive community experience might be leading to word-of-mouth or trust that someone will respond to any concerns, even if not directly related to triatomine bugs, leading to sustained community engagement.

## Conclusions

Surveillance strategies developed decades ago have had limited “update”, despite new technologies and new communication systems in our societies. AlertaChirimacha, an internet-based tool created to improve surveillance and control of urban Chagas disease vectors, has proven more effective than traditional surveillance methods and has great potential for sustainability due to its low human resource demands. The tool engages communities to report triatomines using a streamlined communication approach: instead of taking suspected insects to their health post and waiting for a response, residents can take a picture of the insect and send it via WhatsApp to receive a quick response regarding their concern. The direct line of communication created between residents and vector control authorities did not create an undue burden on the control program, and the dependable and prompt responses generated positive feedback. In a broader context, the AlertaChirmacha system has potential to serve as a valuable source of spatial data, which could be integrated with additional data collected through active surveillance, to better pinpoint high-risk areas for vector infestation. In sum, requiring fewer human and financial resources, in a period of 26 months, more infested households were detected and treated via AlertaChirimacha than the two traditional surveillance methods, suggesting an effective and potentially sustainable approach for control and elimination of hard-to-find vectors.

## Supporting information

S1 Table Community taxon (iNaturalist) of reports. Taxon given by the iNaturalist community based on the visual characteristics of the photos sent by inhabitants when they reported through AlertaChrimacha.
(XLSX)

S2 Table AlertaChirimacha posts on Facebook. General information, link, and results of each AlertaChirimacha Facebook add.
(XLSX)

S3 Table Data base of AlertaChirimacha Reports. Data base of AlertaChirimacha reports received for 26 months. Links to iNaturalist registry is provided.
(XLSX)

S1 Text Spanish version of the manuscript. (DOCX)
