# Supplementary material for: An effective internet-based system for surveillance and elimination of triatomine insects: AlertaChirimacha
Source: PLoS Negl Trop Dis. 2023 Oct 16;17(10):e0011694. doi: 10.1371/journal.pntd.0011694 (PMC10602375; doi:10.1371/journal.pntd.0011694)
Supplement: S1 Text — (DOCX) [file pntd.0011694.s004.docx]

Un sistema eficaz basado en Internet para la vigilancia y eliminación de insectos triatominos: AlertaChirimacha

# Resumen

Las enfermedades transmitidas por vectores siguen siendo una amenaza significativa para la salud pública en muchas regiones del mundo. Los métodos tradicionales de vigilancia y control de vectores han dependido de programas de vigilancia activa y pasiva, que a menudo son costosos y requieren mucho tiempo. Los nuevos sistemas de vigilancia de vectores basados en Internet han mostrado ser prometedores al reducir parte de la carga económica y trabajo para las autoridades de salud. Desarrollamos y evaluamos la efectividad de un nuevo sistema de vigilancia basado en Internet, "AlertaChirimacha", para detectar *Triatoma infestans* (conocido localmente por su nombre quechua, Chirimacha), el vector de la enfermedad de Chagas, en la ciudad de Arequipa, Perú. En los primeros 26 meses después de la implementación, AlertaChirimacha recibió 206 reportes de residentes que sospechaban o temían la presencia de triatominos en sus hogares o vecindarios, de los cuales confirmamos, a través de imágenes o inspecciones, 11 (5.3%) de ellos. Después de un examen microscópico, ninguno de los especímenes recolectados estaba infectado con *Trypanosoma cruzi.* AlertaChirimacha recibió un 57% más de reportes confirmados que el sistema de vigilancia tradicional y detectó un 10% más de viviendas infestadas que los enfoques de vigilancia activa y pasiva combinados. A través de entrevistas en profundidad, evaluamos el alcance, la participación bilateral, y la rapidez y eficiencia de respuesta de AlertaChirimacha. Nuestro estudio destaca el potencial de los sistemas de vigilancia de vectores basados en Internet, como AlertaChirimacha, para mejorar los esfuerzos de vigilancia y control de vectores en entornos con recursos limitados. Este enfoque podría reducir el costo y el tiempo necesario para la eliminación de la enfermedad de Chagas transmitida por vectores en la región.

# Resumen del autor

# Los insectos que transmiten enfermedades siguen siendo un problema en muchas partes del mundo. Monitorearlos y controlarlos puede ser costoso y tomar mucho tiempo, pero nuevos métodos basados en Internet han mostrado ser prometedores para reducir la carga de trabajo y los costos. Desarrollamos AlertaChirimacha, un sistema de vigilancia basado en Internet para detectar a *Triatoma infestans*, una especie de insecto vector de la enfermedad de Chagas. AlertaChirimacha se probó en Arequipa, Perú, y en los 26 meses posteriores a su implementación, recibió 206 informes de residentes que sospechaban tener estos insectos en sus hogares, de los cuales 11 fueron confirmados por nuestro equipo. AlertaChirimacha detectó más viviendas infestadas que los métodos tradicionales combinados. AlertaChirimacha también fue efectivo en llegar a las personas, lograr su participación y recibir respuestas a los reportes. Este enfoque puede mejorar los esfuerzos para controlar enfermedades transmitidas por insectos en lugares con recursos limitados y lograr que sea menos costoso eliminar la enfermedad de Chagas transmitida por vectores.

# **Palabras clave:** e-health, vectores de enfermedades infecciosas, vigilancia, Triatominae

# Introducción

La vigilancia y el control de vectores han sido la principal, y en algunos casos, la única estrategia para reducir la morbilidad y mortalidad causada por enfermedades transmitidas por vectores [1]. Los programas de vigilancia vectorial tradicionalmente se han basado en dos componentes: la vigilancia activa, que requiere de búsquedas físicas realizadas por personal de salud, y la vigilancia pasiva, que se basa en los reportes de personas afectadas. La vigilancia activa es costosa y lleva mucho tiempo, pero generalmente puede proporcionar información precisa para la detección temprana y la prevención de brotes [2]. La vigilancia pasiva es menos costosa (para las autoridades de salud) y puede proporcionar datos útiles, pero requiere un sistema eficiente para la recepción de información y una respuesta rápida. Ambos enfoques juntos a menudo son más efectivos que la suma de sus partes [3].

El vector de la enfermedad de Chagas, Triatoma *infestans*, ha sido controlado mediante la aplicación de insecticidas residuales en interiores en varias regiones del sur de Sudamérica [4] a través de un programa coordinado conocido como la Iniciativa del Cono Sur (INCOSUR) [5–7]. El seguimiento entomológico continuo después de estos éxitos, combinado con un control extenso y rápido, es esencial para evitar la reaparición del insecto. La ciudad de Arequipa planteó uno de los mayores desafíos para la Iniciativa del Cono Sur, ya que el vector se había dispersado ampliamente en el denso entorno urbano [8,9], pero se ha logrado un gran progreso en los últimos años en la reducción de la infestación en los hogares. Sin embargo, a medida que los programas de control se acercan a la eliminación de la transmisión de *Trypanosoma cruzi* transmitido por vectores, se vuelve más difícil mantener una vigilancia efectiva, ya que se convierte en una preocupación de salud menos prioritaria y los recursos humanos y financieros se asignan a otros programas.

El dicho de "buscar una aguja en un pajar" es apropiado: Menos vectores son más difíciles de encontrar, dando como resultado una disminución del riesgo percibido a nivel comunitario, una disminución en la motivación de los inspectores y profesionales de la salud para buscar al vector, y una reducción de la atención y de los recursos/presupuestos asignados por un sistema de salud con múltiples prioridades [10–13]. Los nuevos sistemas de vigilancia de vectores basados en Internet tienen un gran potencial para fortalecer la vigilancia y aliviar parte de la carga económica y laboral de las autoridades de salud [14,15]. Estos sistemas han sido diseñados y probados en diferentes países, con diversos grados de éxito. Algunos programas han tenido éxito en la participación comunitaria para reportar acerca de vectores a través de aplicaciones o sistemas basados en Internet [16–21]. Varios programas también han descrito la importancia de la participación de la comunidad en el proceso de control de vectores – un proceso que crea y construye relaciones y permite el intercambio de información entre profesionales de la salud pública y miembros de la comunidad [19,22,23]. Esta participación bilateral ayuda en la recopilación de datos y en la identificación de focos de vectores al promover una mayor educación y conciencia entre los miembros de la comunidad [18,23]. Respuestas oportunas y profesionales a preguntas o mensajes de la comunidad sobre posibles infestaciones son clave para demostrar que la participación de la comunidad es valorada y que la vigilancia y el control de vectores requiere que todos trabajen como parte de un sistema más amplio [13], así como herramientas para reducir las barreras en la comunicación y el reporte. Las respuestas rápidas a los miembros de la comunidad los mantiene comprometidos con el proceso y fomenta una mayor acción. En última instancia, se ha descrito que estos programas tienen un amplio alcance en la población y reducen los costos para el sistema de salud [16].

En respuesta a los desafíos anteriormente mencionados para el sistema de vigilancia de triatominos, combinados con los confinamientos debido a la Covid, desarrollamos un sistema de vigilancia y respuesta de triatominos basado en Internet para la ciudad de Arequipa: "AlertaChirimacha" para la detección de *Triatoma infestans*, un insecto triatomino (conocido localmente por su nombre quechua, Chirimacha). Alejándonos de un sistema tradicional jerárquico de vigilancia y control de vectores, AlertaChirimacha está diseñado para facilitar un programa integrado de vigilancia y control de vectores más eficiente y coordinado [24]**.**

# Métodos

## Declaración Ética

El protocolo de investigación fue aprobado por los comités de ética de la Universidad Peruana Cayetano Heredia (Número de aprobación: 103096) y de la Universidad de Pensilvania (Número de aprobación: 833122). Solo se recopilaron datos entomológicos.

## Sitio de estudio

AlertaChirimacha se lanzó en Arequipa, una ciudad andina con aproximadamente 1 millón de habitantes, ubicada a 2,300 metros sobre el nivel del mar en el sur de Perú [25]. Una campaña de control de triatominos (a partir de aquí denominada "campaña") llevada a cabo entre 2003 y 2018 casi ha logrado eliminar a *Triatoma infestans* de los distritos afectados. Después de la campaña, se implementó un sistema de vigilancia convencional de manera gradual tras la aplicación de insecticidas en cada distrito [26]. El sistema convencional incluye tanto la vigilancia pasiva como activa. El sistema de vigilancia pasiva requiere que los residentes que sospechan tener triatominos en su hogar capturen un insecto y lo lleven a un puesto de salud para su identificación. Si se confirma que el insecto es *Triatoma infestans*, las autoridades de salud regionales o nuestro personal realizan una inspección de la vivienda que reportó el insecto y de las viviendas adyacentes, expandiéndose hacia afuera hasta encontrar dos viviendas consecutivas donde no se detecte la presencia de insectos triatominos. Las viviendas infestadas y sus vecinos inmediatos son tratados con insecticida, se educa a los residentes de la zona y se les hace conscientes de la necesidad de estar alerta ante este insecto. AlertaChirimacha se lanzó por primera vez en octubre de 2020; aquí informamos los resultados de los primeros 26 meses (hasta diciembre de 2022).

## AlertaChirimacha

AlertaChirimacha fue diseñado para funcionar a través de tres mecanismos principales: 1) un **alcance** (cobertura) amplio y económico en los hogares para comunicar mensajes educativos, 2) un **compromiso bilateral** entre las autoridades de salud, nuestro equipo de investigación y la comunidad para permitir preguntas y respuestas, así como la difusión de información específica, y 3) una **respuesta rápida y eficiente** a la notificación de vectores por parte de la comunidad.

**Alcance:** A través de un trabajo piloto formativo, determinamos que Facebook y WhatsApp son ampliamente utilizados en nuestra población, y diseñamos un video educativo (Video S1). El video tiene dos objetivos: 1) Clarificar los procesos de reporte que la población puede utilizar y 2) Educar a la población sobre cómo luce *T. infestans* (o sus manchas secas de heces en un hogar), dónde es más probable que se encuentre, así como información sobre la enfermedad de Chagas. Pagamos por publicaciones, aproximadamente 50 USD por anuncio, que se lanzaron cada 3 meses en toda la región de Arequipa. La página de Facebook estaba vinculada a las dos páginas de Facebook de la Gerencia Regional de Salud y la Red de Salud Arequipa-Caylloma (equivalentes al Ministerio de Salud Regional de Arequipa y al Sistema de Salud Regional) con 174,000 y 11,000 seguidores respectivamente (Figura 1).

**
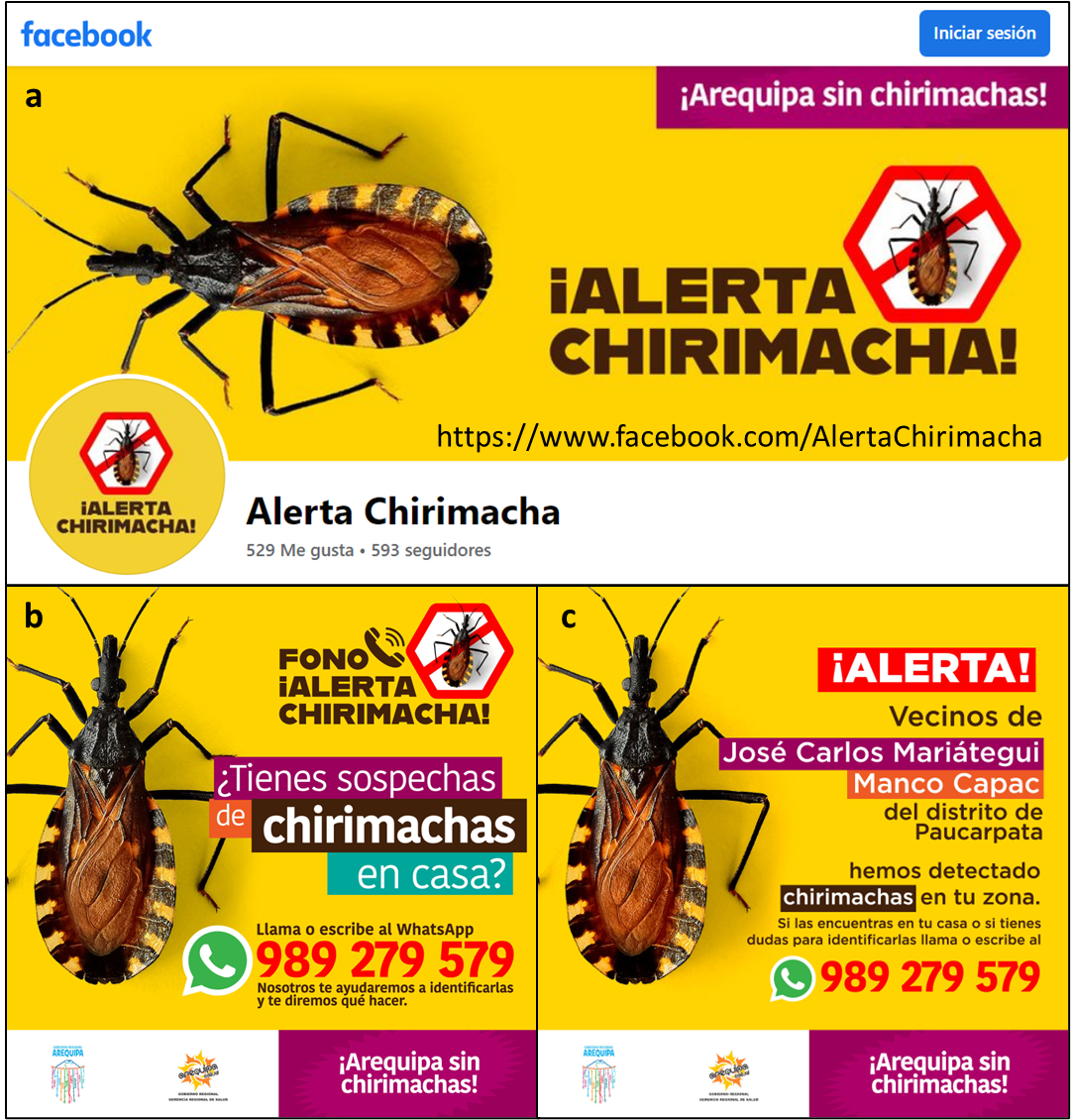
**

**Fig 1. Página de Facebook y publicaciones de AlertaChirimacha . a**. Página de inicio de AlertaChirimacha en Facebook (<https://www.facebook.com/AlertaChirimacha>). **b**. Diseño de la primera publicación, promovida a través de Facebook y con el uso de volantes, con el objetivo de informar a la comunidad sobre el nuevo sistema y el número de contacto. **c**. Diseño de una publicación específica dirigida a informar a la población que se han encontrado chirimachas (nombre local para los triatominos) en su vecindario y pidiéndoles que busquen y reporten si encuentran alguno en sus hogares.

Para garantizar un amplio alcance entre aquellos que no utilizan Facebook (por ejemplo, las personas mayores), también promovimos el uso del WhatsApp de AlertaChirimacha para reportar posibles triatominos a través de volantes y afiches: algunos eran pegados en los centros de salud, algunos en bodegas, otros fueron distribuidos puerta a puerta por especialistas en control vectorial durante su trabajo de vigilancia activa y otros volantes se entregaron en los vecindarios ubicados a menos de 200 metros de zonas donde se reportó una infestación.

**Compromiso bilateral:** la participación de la comunidad fue fundamental en AlertaChirimacha, y para ello, era importante crear un sistema que permitiera la comunicación bilateral; en última instancia, esto generaría un sistema dinámico al que los miembros de la comunidad pudieran acudir para resolver preguntas rápidamente, así como generar confianza en la experiencia de quienes respondían a las preguntas de manera exhaustiva. Por lo tanto, un biólogo revisaba y respondía de manera exhaustiva a todos los comentarios en el sitio de AlertaChirimacha en un plazo de 2 a 3 días. En todas las comunicaciones difundidas (anuncios, volantes y carteles), también promovimos un número de teléfono al que las personas podían llamar o enviarnos mensajes directamente: para enviar fotos, hacer más preguntas o informar sobre insectos sospechosos. Los residentes podían enviar fotos del insecto o de signos de su presencia; si los residentes no podían proporcionar una foto, podían describir lo que habían visto. Por cada foto recibida, el biólogo confirmaba la especie y respondía. En el caso de *T. infestans*, se enviaba a un especialista en control de vectores para realizar una inspección exhaustiva de la vivienda y de sus vecinos cercanos, como se describe anteriormente. En el caso de que el insecto reportado no sea insecto objetivo, el biólogo respondía identificando la especie del insecto, brindando tranquilidad de que no era peligroso y también explicando, generalmente con imágenes, cómo difiere el insecto reportado de *T. infestans*. Además, si en la foto se identificaba que el insecto reportado era un chinche de cama (género de *Cimex*), el biólogo enviaba un folleto informativo [La versión en español de "Preventing and Getting Rid of Bed Bugs Safely" del Departamento de Salud y Salud Mental de Nueva York]. [27], y respondía preguntas relacionadas.

Para los reportes sin fotografía, el biólogo realizaba más preguntas relacionadas con las características del insecto, incluyendo el lugar y la hora del día en el que se encontró el insecto. En algunos casos, la descripción era suficiente para descartar que fueran triatominos, como en el caso de insectos encontrados entre plantas de jardín (extremadamente improbable para *T. infestans*). Se le informaba a la persona sobre qué podría ser el insecto y se le pedía que intentara tomar una foto del insecto si lo volvían a encontrar. Sin embargo, si había alguna duda, se enviaba a un especialista en control de vectores para inspeccionar la vivienda.

**Respuesta rápida y eficiente:** El sistema pasivo convencional de reportes tiene múltiples puntos en los que se puede perder información crítica. Hay un control de calidad limitado dentro del sistema para garantizar que los residentes que logran atrapar un insecto en una bolsa y llevarlo a un puesto de salud reciban inspecciones de seguimiento y tratamiento con insecticidas cuando sea necesario. Actualmente, es imposible calcular el número real de triatominos que nunca fueron informados o que fueron informados y nunca registrados por el sistema de salud. Por lo tanto, después de la confirmación de triatominos, ya sea por foto o inspección, nuestro equipo de investigación, en coordinación con el Ministerio de Salud Regional de Arequipa, enviaba un equipo de control de vectores a la vivienda informante en un plazo de 7 días para realizar inspecciones. Los hogares en los que se identificaba al menos un vector de la enfermedad de Chagas, independientemente de su estado de infección por *Trypanosoma cruzi*, recibían tratamiento con insecticida residual siguiendo los protocolos establecidos por el Ministerio de Salud del Perú. Todas las muestras, excepto las ninfas de primer estadio eran examinadas para detectar la presencia de *Trypanosoma cruzi*. Para este propósito, se comprimía suavemente en el área abdominal de cada insecto para extraer gotas de heces. Estas gotas se diluían con una gota de solución salina al 0.9% y se colocan bajo una lámina de microscopio de 22x22. Posteriormente, la muestra se observaba bajo una magnificación de 400x para detectar tripanosomas activos [28]. Además, en el contexto de un ensayo aleatorizado por conglomerados más amplio en el que se enarca este trabajo, [24], cuando se detectaba un triatomino en zonas de la ciudad asignadas al área de intervención, designábamos un área de 200 metros alrededor de la vivienda infestada para activar el sistema de vigilancia con más intensidad. Dentro de esta zona de intervención, hacíamos lo siguiente: 1) visitábamos todas las viviendas, realizábamos inspecciones y distribuíamos volantes que promocionaban AlertaChirimacha. 2) Colocábamos afiches en todas las tiendas que estuvieran dispuestas a permitirlo 3) Creamos y promocionamos publicaciones específicas en Facebook durante 10 días para los residentes que vivían dentro del radio de 1 km de la vivienda infestada (el área más pequeña que se podía crear para estas publicaciones en Facebook, y este rango mínimo cambiaba con el tiempo debido a las regulaciones de Facebook). Esta intervención tenía como objetivo informar que se había encontrado una infestación en la zona y alentar a los residentes de la zona a inspeccionar sus hogares en busca de triatominos y a informar cualquier hallazgo de un insecto sospechoso o de sus signos (manchas secas de heces) a través de WhatsApp o una llamada telefónica (Figura 1C). Si encontrábamos viviendas adicionales infestadas cerca del foco original, se publicaba un segundo mensaje a los 10 y 30 días después del mensaje inicial. Los mensajes posteriores informaban a los vecinos que se habían encontrado más viviendas positivas y les pedían que se mantuvieran alerta ante la presencia de triatominos y que los informaran si los encontraban. Es imposible mantener a una comunidad en estado de alerta constante indefinidamente, por lo tanto, una vez que no encontrábamos viviendas adicionales infestadas, publicábamos un mensaje 30 días después de la última publicación, explicando que las viviendas afectadas habían sido tratadas con insecticidas y que la infestación había sido controlada.

## Análisis

Se utilizaron varias métricas para evaluar el éxito de los tres mecanismos. Para evaluar el alcance de AlertaChirimacha, utilizamos las métricas de Facebook : "alcance" (número de personas que vieron el anuncio al menos una vez), el número total de reacciones, comentarios, veces que se compartieron las publicaciones y veces que se hizo clic en ellas para cada anuncio publicado. También evaluamos el alcance a través del número de carteles aún visibles en las tiendas locales 90 días después de ser colocados. Además, según los datos recopilados al momento de reportar sobre los insectos a nuestro equipo a través de llamadas telefónicas o WhatsApp, presentamos el número de viviendas que recibieron un folleto de AlertaChirimacha. Con respecto al compromiso bilateral, todas las preguntas y comentarios en Facebook se documentaron en una base de datos de Excel, así como la respuesta dadas por nuestro biólogo a cada pregunta o comentario. Revisamos todas las preguntas y comentarios, y codificamos de manera inductiva todos los comentarios en cuatro temas principales: 1) preguntas o información sobre el vector, la enfermedad o el sistema de informes, 2) reportes sobre el vector, 3) expresiones de miedo, preocupación o agradecimiento con respecto a la información proporcionada, y 4) otros temas aleatorios. Los hallazgos se presentan en una gráfica, junto con citas que representan los tipos de comentarios de las tres categorías principales. En cuanto a la respuesta rápida y eficiente, describimos el tiempo promedio de respuesta entre la recepción de una queja positiva, la inspección y tratamiento.

# Resultados

Todos los resultados se basan en los hallazgos de los primeros 26 meses posteriores a la implementación de AlertaChirimacha. Recibimos 206 reportes de residentes que sospechaban o temían la presencia de triatominos en sus hogares o vecindarios en Arequipa. La mayoría de los reportes fueron sobre insectos que no eran triatominos (Figura 2), como un insecto fitófago del género Vazquezitocoris (23%) (Figura 3). Once (5.3%) de los reportes recibidos en Arequipa fueron confirmados como *Triatoma infestans*. También recibimos 24 reportes de 15 provincias fuera de Arequipa, tres de los cuales se confirmaron como vectores de la enfermedad de Chagas (dos eran *Panstrongylus chinai* y uno era un miembro del género *Triatoma* que no pudo identificarse completamente a partir de la fotografía del informe, consulte la Tabla S1). Recibimos 19 reportes adicionales que no indicaban la ubicación. Es importante destacar que se detectaron y confirmaron más viviendas infestadas a través de AlertaChirimacha (11) que mediante los reportes de vigilancia tradicionales durante el mismo periodo: vigilancia pasiva (7) y vigilancia activa dirigida por especialistas en control vectorial (3, de un total de 9,574 inspecciones realizadas por personal capacitado durante ese período). Ninguno de los insectos examinados estaba infectado con *Trypanosoma cruzi*. Lamentablemente, los insectos vectores relacionados con reportes de provincias fuera de Arequipa no pudieron examinarse para detectar la presencia de *T. cruzi* debido a complicaciones logísticas para transportar las muestras a nuestro laboratorio en Arequipa.


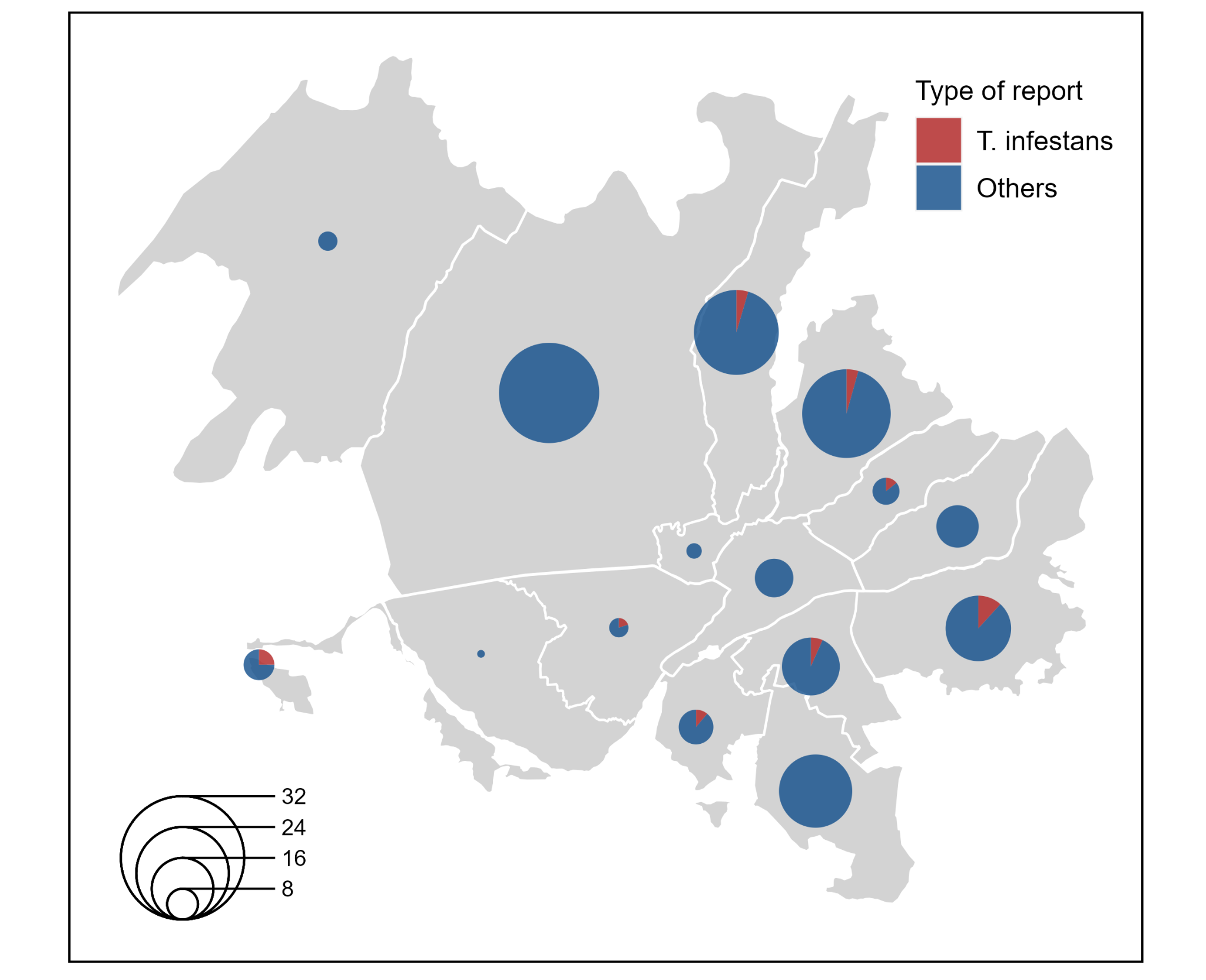


**Fig 2**. **Distribución de los reportes de AlertaChirimacha por distrito en el área metropolitana de Arequipa, Perú.** Los gráficos circulares representan el número total de reportes; las secciones en rojo representan los reportes confirmados como *Triatoma infestans*.

**Alcance:** En 26 meses, publicamos 32 anuncios en Facebook: 6 fueron publicaciones generales sobre la enfermedad de Chagas y su vector. Además, un total de 4,313 hogares recibieron información sobre AlertaChirimacha a través de campañas de comunicación puerta a puerta. Nuestra primera publicación en Facebook, un folleto simple que presentaba AlertaChirimacha (Figura 1), tuvo un alcance relativamente pequeño (2,528), pero fue la publicación más compartida entre los usuarios de Facebook (653). En las publicaciones posteriores, el alcance aumentó significativamente, alcanzando su punto máximo en 60,028 para la última publicación. Sin embargo, a medida que aumentó el número de publicaciones, la cantidad de compartidos disminuyó (Figura 4). Por otro lado, las publicaciones de video tuvieron un gran alcance (34,368 y 59,287) pero no generaron mucha interacción. No hubo una tendencia en las reacciones o en los “compartido” de publicaciones específicas a lo largo del tiempo (consulte la Tabla S2).


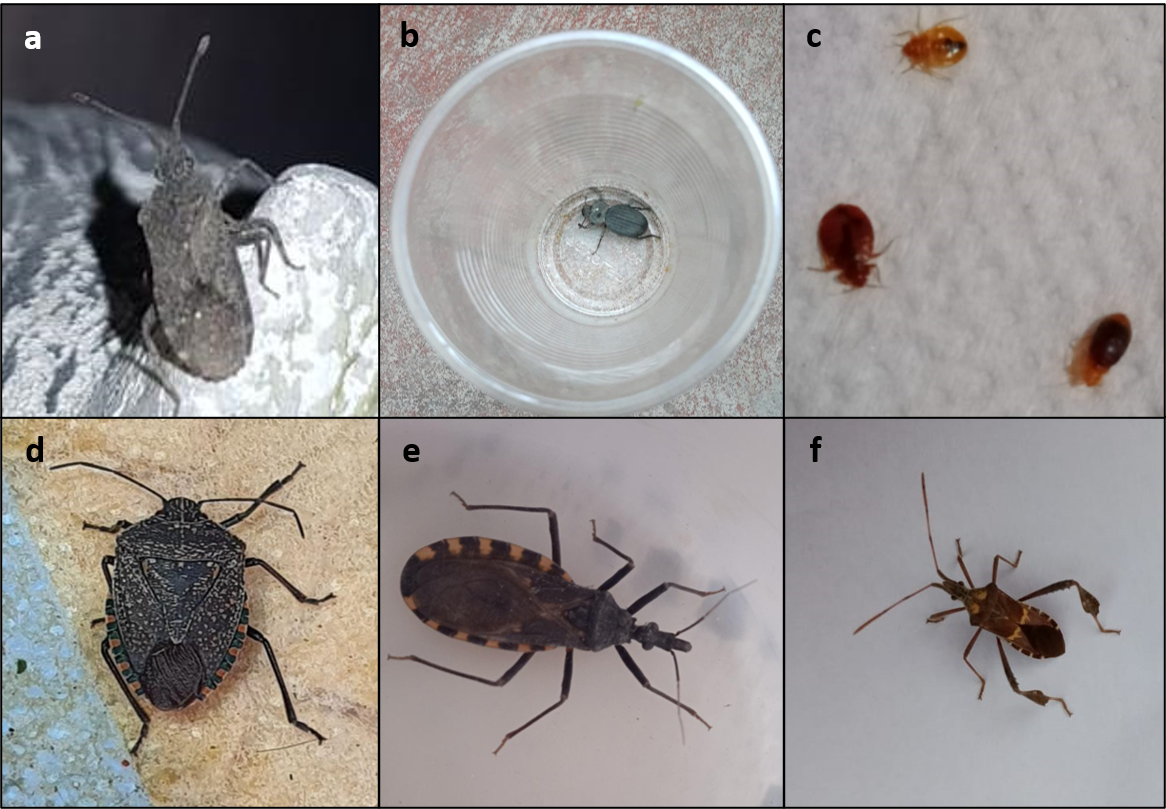


**Fig 3**. **Los 6 insectos más comúnmente reportados a través de AlertaChirimacha.** a. *Vazquezitocoris* sp. b. Coleoptera (Orden) c. Chinches de cama (*Cimex sp*) d. *Pellaea stictica* e. Chirimacha (*Triatoma infestans)*, f. *Leptoglossus zonatus*. Una lista completa de los reportes se encuentra recopilada en iNaturalist con enlaces proporcionados en la Tabla S3.

En el radio de 200 metros alrededor de las infestaciones, 395 de las 400 tiendas aceptaron tener un cartel pegado en la pared. Aquellos que se negaron indicaron que no tenían "espacio" en su pared o que no querían carteles en su pared. Cuando se volvió a visitar las tiendas 90 días después de completar el trabajo, aproximadamente el 85% de estos carteles todavía eran visibles para la población; el otro 15% estaba cubierto por anuncios de productos o simplemente se había retirado. Esta difusión de información e inspecciones dentro del radio de 200 metros, combinada con las publicaciones dirigidas en Facebook, nos permitió detectar 5 viviendas adicionales infestadas además de las que se habrían detectado si solo se hubiera seguido el protocolo tradicional del Ministerio de Salud Regional (inspeccionar viviendas adyacentes hasta que los inspectores lleguen a dos viviendas negativas consecutivas).


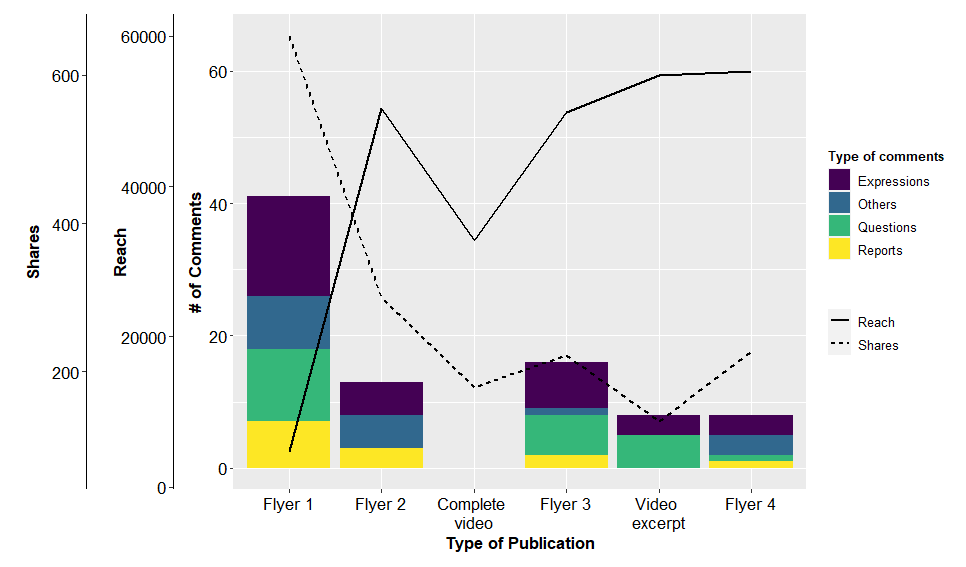


**Fig 4.** **Métricas de Facebook y comentarios para las publicaciones de AlertaChirimacha.** El alcance (número de personas expuestas a la publicación), los compartidos (número de personas que compartieron la publicación) y los comentarios (categorizados como expresiones, preguntas, informes y otros) para las publicaciones de AlertaChirimacha entre octubre de 2020 y diciembre de 2022.

**Compromiso bilateral:** La mayoría de los comentarios en las publicaciones eran preguntas relacionadas con el vector o la enfermedad, como: por qué el triatomino es peligroso, dónde vive y cómo evitar que entre en las viviendas. Otros comentarios frecuentes en AlertaChirimacha provenían de personas que añadían información sobre los triatominos y la enfermedad de Chagas, ocasionalmente describiendo sus experiencias pasadas con los vectores o la enfermedad. Por ejemplo, algunos comentarios venían de migrantes venezolanos que reconocían los insectos y mencionaban el nombre común de vectores de la enfermedad de Chagas en su país.

*“Mal de Chagas es la enfermedad, pero su picadura [triatomino] no la produce lo que la produce son sus heces y orina. En caso de picadura no frotarla por que implicaría mayor riesgo de usted mismo infectarse llevando con su mano la orina o heces a la herida. Otro dato importante en caso de picadura es no matar al insecto; lo contrario debe atraparlo vivo y así poder llevarlo a su análisis para saber si está contaminado o no lo está para su tranquilidad, ya que la enfermedad da positivo años más tarde desde su contagio”*

*“En mi país se llama chipo [triatomino] y da mal de Chagas eso es inflamación del corazón, pero da después de los 50 años. Si lo llegan a ver no lo maten porque su líquido que bota igual hace daño”.*

*“Estos bichos tienen alas, son de olor horrible, de color oscuro, un poco largos, se esconden en lugares oscuros, bajo las sillas”.*

Además, hubo expresiones -tanto en texto como a través de emojis- de alarma, miedo y preocupación, así como de aprecio por el sitio. Varias personas etiquetaron a sus amigos para difundir el mensaje. Algunas personas utilizaron los comentarios para informar que habían visto el insecto o sugerir áreas donde podría encontrarse; sin embargo, estos informes sobre "otros" generalmente se referían a lugares reconocidos como desordenados, sucios o con presencia de animales.

“*Mi vecino vive como en un basurero. No tiene muro de contención y allí hecha su basura. Él dice que tiene mucho dinero, pero no hace su muro… más que seguro que también tiene chirimachas ¿a quién puedo acudir?”*

También hubo una cantidad limitada de comentarios que difundieron información falsa, como decir que los insectos son inofensivos o incluso que "no existen". Abordamos estos comentarios caso por caso, eliminando algunos comentarios que eran políticos u ofensivos, y pasando por alto otros. Observamos que estos comentarios contraproducentes no ganaron relevancia y recibieron pocos o ningún "me gusta" o reenvío.

**Respuesta rápida y eficiente:** Se han tratado un total de 68 viviendas (20 infestadas y 48 consideradas de riesgo por su proximidad a viviendas infestadas) en respuesta a la detección de viviendas infestadas a través de AlertaChirimacha. Hemos podido responder en un plazo de 1 a 3 días a todos los reportes de insectos a través de AlertaChirimacha. En el caso de los reportes positivos, todos los hogares fueron inspeccionados en el plazo de una semana a partir del reporte, y todos los hogares infestados fueron tratados inmediatamente después de la confirmación (aunque experimentamos algunos retrasos debido a las lluvias, la pandemia y las protestas políticas). No se puede estimar la tasa de respuesta y el tiempo de respuesta a través del sistema de notificación tradicional, ya que muchos reportes se pierden en el proceso y no siempre se anotan las fechas de los reportes. En algunas ocasiones, las personas que reportaron triatominos a través de AlertaChirimacha señalaron que ya habían reportado el insecto con anterioridad a través del sistema tradicional y no habían recibido respuesta, en algunos casos incluso seis meses después; de ahí su decisión de ponerse en contacto con este número.

# Discusión

AlertaChirimacha, surgida de la necesidad durante los confinamientos por la pandemia en Perú, se ha convertido en una herramienta fundamental para la vigilancia y el control de los vectores urbanos de la enfermedad de Chagas. A lo largo de dos años, se detectaron y trataron más viviendas infestadas a través de AlertaChirimacha que mediante los dos métodos tradicionales de vigilancia: la búsqueda activa realizada por especialistas en control vectorial y la recepción pasiva de insectos en los centros de salud, combinados. Esta herramienta también tiene un gran potencial de sostenibilidad debido a su baja demanda de recursos humanos y, si la tasa de detección de viviendas infestadas continúa superando a los métodos existentes, podría reducir el costo y el horizonte temporal para la eliminación de la enfermedad de Chagas mediada por vectores en la región.

En el contexto epidemiológico de Arequipa, donde la eliminación del único vector de la enfermedad de Chagas está cerca, los focos identificados en el transcurso de 26 meses no son desdeñables. Barbu et al. estimaron una prevalencia residual de infestación después de la aplicación de insecticidas en los distritos tratados del 1.2% [26]; a lo largo de los años sucesivos, se han identificado y eliminado focos residuales re-emergentes, lo que ha resultado en una reducción adicional de la prevalencia. Esto, a su vez, ha llevado a una disminución en el número de reportes recibidos, con un promedio de 32 reportes por año en los tres años previos a la implementación de AlertaChirimacha. Por lo tanto, la cantidad de reportes recibidos y los focos descubiertos a través de AlertaChirimacha constituyen una evidencia convincente de que el sistema sigue siendo efectivo incluso en presencia de una baja tasa de infestación.

El objetivo de AlertaChirimacha era mejorar la vigilancia al involucrar a la comunidad en el reporte de la presencia de triatominos utilizando un enfoque de comunicación simplificado que fuera factible desde casa y durante un confinamiento. Se presentaron muchos desafíos para el sistema tradicional de vigilancia y control: se requería que los residentes capturaran un insecto peligroso en una bolsa y lo llevaran a un centro de salud, y una vez allí, se esperaba que los residentes navegaran por la jerarquía del centro de salud para asegurarse de que el insecto llegara a las manos adecuadas (por ejemplo, el especialista en control vectorial). Incluso si lo hacían, no había garantía de que el reporte llegara a las autoridades de control de vectores del Ministerio de Salud de la Región, que supervisa a los técnicos de control de vectores y asigna insecticidas. En cambio, AlertaChirimacha creó una línea directa de comunicación entre los residentes y las autoridades de control de vectores de la GERESA (Ministerio de Salud de la Región). También integró la presentación de reportes, inspecciones confirmatorias y tratamiento; por lo tanto, eliminó lagunas en el flujo de información y facilitó respuestas rápidas y eficientes.

AlertaChirimacha requirió un cambio de comportamiento por parte de la población: pasar de llevar insectos sospechosos a su centro de salud a tomar una foto del insecto y enviarla por WhatsApp o Facebook Messenger. El Modelo del Cambio de Comportamiento es un modelo teórico que describe que el cambio de comportamiento requiere intervenciones que se centren en desarrollar la capacidad, proporcionar oportunidades para el cambio y aumentar la motivación (COM-B) - siendo todos componentes importantes de AlertaChirimacha [29]. A través de los mensajes y videos de Facebook, construimos la capacidad de la comunidad para identificar al triatomino y saber dónde buscarlo, proporcionamos oportunidades factibles para informar de manera simplificada a través de WhatsApp en sus teléfonos y Facebook Messenger, y al garantizar una respuesta rápida y oportuna, aumentamos la motivación para informar. Además, AlertaChirimacha fue diseñada utilizando principios fundamentales de educación y promoción de la salud comunitaria, como la participación de la comunidad y la priorización de las preocupaciones de la comunidad (respondiendo y proporcionando información sobre todas las imágenes de insectos que les preocupaban) [30]. Otros programas de reporte de vectores basados en Internet se han implementado para garrapatas, mosquitos e insectos triatominos. [16–19,21,31], y, en contraste con AlertaChirimacha, muchos de estos programas han enfrentado desafíos para lograr un uso sostenido por parte de la comunidad o la integración de datos [21].

La línea directa de comunicación con el equipo de investigación y las autoridades de salud encargadas de supervisar la aplicación de insecticidas no creó una carga excesiva en el programa de control. El número de informes recibidos fue grande, pero estos fueron respondidos fácil y rápidamente (por LDTQ o CEC). Todos los que informaron sobre cualquier tipo de insecto recibieron una respuesta a su preocupación, ya sea que se tratara de un triatomino o no. Se creó así un ciclo de retroalimentación positiva, donde las personas aprendieron que los informes se tomaban en serio y se respondían rápidamente. Sospechamos que esto, más que cualquier otra cosa, llevó al uso sostenido del Sistema [13]. La capacidad de compartir imágenes de insectos también aumentó la eficiencia, tanto para la comunidad como para el sistema. Pudimos calmar los temores y solventar todas las consultas a través de mensajes de texto, evitando cientos de inspecciones innecesarias. Las respuestas rápidas y estructuradas no solo se proporcionaron a quienes presentaron reportes, sino que también fueron recibidas por quienes hicieron consultas y comentarios en las publicaciones de Facebook. Esto no solo hizo que los usuarios sintieran que su participación era valorada, sino que también ayudó a otros usuarios de Facebook a confiar en el sistema.

Nuestro sistema de informes y nuestro estudio tienen limitaciones. Si bien el acceso a teléfonos inteligentes y Facebook es extremadamente alto en Perú [32,33], es probable que las personas mayores que no estén tan conectadas a las redes sociales y las poblaciones más marginadas que puedan tener menos acceso digital estuvieran menos informadas. La inclusión de la distribución de folletos físicos y la colocación de carteles en bodegas de áreas afectadas pueden haber contribuido al éxito general del sistema. Otra limitación es el uso de métricas de reacción de Facebook (es decir, me gusta, compartir) para evaluar el componente "alcance" de AlertaChirimacha, ya que pueden representar a poblaciones fuera del área de estudio o regiones donde la presencia de otros insectos que no son triatominos puede desencadenar respuestas. Del mismo modo, la sensibilidad de la microscopía para la detección de *T. cruz* es imperfecta y es posible que hayamos pasado por alto algunos insectos infectados. Sin embargo, cabe resaltar que no hemos identificado la presencia del parásito en Arequipa urbana desde 2015. También es importante señalar que la naturaleza de los datos reportados no permite hacer estimaciones precisas sobre la prevalencia de la infestación.

Existen muchas implicaciones políticas y programáticas de este trabajo. Aunque se necesita un estudio detallado de costo-efectividad para comparar los recursos invertidos en AlertaChirimacha en comparación con el sistema de vigilancia tradicional, AlertaChirimacha requiere recursos de personal limitados para llegar, relacionarse con los hogares y garantizar una respuesta oportuna a los casos reportados, por lo que es probable que sea mucho más rentable. Utiliza tecnologías que no estaban disponibles hace veinte años que nos permiten "entrar" en decenas de miles de hogares. De hecho, incluso llegó a hogares fuera de la ciudad de Arequipa, lo que demuestra que esta estrategia podría cubrir un territorio mucho más grande y podría usarse potencialmente para otras enfermedades transmitidas por vectores o zoonóticas que requieren reportes de la comunidad, como las garrapatas y la rabia canina. Solo el tiempo nos dirá si esta estrategia mantiene el impulso: nuestros primeros 26 meses han demostrado que la experiencia positiva de la comunidad podría estar llevando a la propagación de boca a boca o la confianza en que alguien responderá a cualquier inquietud, incluso si no está directamente relacionada con los insectos triatominos, lo que lleva a un compromiso comunitario sostenido.

# Conclusiones

Las estrategias de vigilancia desarrolladas hace décadas han tenido una "actualización" limitada, a pesar de las nuevas tecnologías y sistemas de comunicación en nuestras sociedades. AlertaChirimacha, una herramienta basada en Internet creada para mejorar la vigilancia y el control de los vectores urbanos de la enfermedad de Chagas, ha demostrado ser más efectiva que los métodos de vigilancia tradicionales y tiene un gran potencial de sostenibilidad debido a sus bajos requerimientos de recursos humanos. La herramienta involucra a las comunidades para que reporten sobre triatominos utilizando un enfoque de comunicación simplificado: en lugar de llevar los insectos sospechosos a su puesto de salud y esperar una respuesta, los residentes pueden tomar una foto del insecto y enviarla por WhatsApp para recibir una respuesta rápida sobre su inquietud. La línea directa de comunicación creada entre los residentes y las autoridades de control de vectores no creó una carga excesiva en el programa de control y las respuestas confiables y rápidas generaron comentarios positivos. En un contexto más amplio, el sistema AlertaChirmacha tiene el potencial de servir como una fuente valiosa de datos espaciales, que podría integrarse con datos adicionales recopilados a través de la vigilancia activa, para identificar mejor las áreas de alto riesgo de infestación por vectores. En resumen, al requerir menos recursos humanos y financieros, en un período de 26 meses, se detectaron y trataron más hogares infestados a través de AlertaChirimacha que mediante los dos métodos de vigilancia tradicionales, lo que sugiere un enfoque efectivo y potencialmente sostenible para el control y la eliminación de vectores difíciles de encontrar.

# Agradecimientos

# Los autores agradecen sinceramente las contribuciones del Ministerio de Salud del Perú (MINSA), la Gerencia Regional de Salud de Arequipa (GRSA) y la Red de Salud Arequipa - Caylloma. Reconocemos el trabajo del equipo de comunicaciones de la ONG Prisma por su contribución en el desarrollo del material educativo para AlertaChirimacha.

# Referencias

1. Wilson AL, Courtenay O, Kelly-Hope LA, Scott TW, Takken W, Torr SJ, et al. The importance of vector control for the control and elimination of vector-borne diseases. PLoS Neglected Tropical Diseases. 2020. doi:10.1371/journal.pntd.0007831

2. Nsubuga P, White ME, Thacker SB, Anderson MA, Blount SB, Broome C V., et al. Public Health Surveillance: A Tool for Targeting and Monitoring Interventions. 2nd ed. Washington, DC: Oxford University Press; 2006.

3. Kalluri S, Gilruth P, Rogers D, Szczur M. Surveillance of arthropod vector-borne infectious diseases using remote sensing techniques: A review. PLoS Pathog. 2007;3: 1361–1371. doi:10.1371/journal.ppat.0030116

4. Silveira AC, Rojas A, Segura E, Guillen G, Russomando G, Schenone H, et al. El control de la enfermedad de Chagas en los países del Cono Sur de América. Historia de una iniciativa internacional 1991/2001. Facultad de Medicina del Triángulo Mineiro; 2002.

5. Schofield CJ, Dias JCP. A cost-benefit analisys of chagas disease control. Mem Inst Oswaldo Cruz. 1991;86: 285–295. doi:10.1590/S0074-02761991000300002

6. Dias JCP. Southern Cone Initiative for the elimination of domestic populations of Triatoma infestans and the interruption of transfusion Chagas disease: historical aspects, present situation, and perspectives. Mem Inst Oswaldo Cruz. 2007;102: 11–18. doi:10.1590/S0074-02762007005000092

7. Coura JR. Chagas disease: control, elimination and eradication. Is it possible? Mem Inst Oswaldo Cruz. 2013;108: 962–967. doi:10.1590/0074-0276130565

8. Levy MZ, Bowman NM, Kawai V, Waller LA, Del Carpio JGC, Benzaquen EC, et al. Periurban Trypanosoma cruzi-infected Triatoma infestans, Arequipa, Peru. Emerg Infect Dis. 2006;12: 1345–1352. doi:10.3201/eid1209.051662

9. Delgado S, Ernst KC, Pumahuanca ML, Yool SR, Comrie AC, Sterling CR, et al. A country bug in the city: urban infestation by the Chagas disease vector Triatoma infestans in Arequipa, Peru. Int J Health Geogr. 2013;12: 48. doi:10.1186/1476-072X-12-48

10. Vazquez-Prokopec GM, Spillmann C, Zaidenberg M, Kitron U, Gürtler RE. Cost-Effectiveness of Chagas Disease Vector Control Strategies in Northwestern Argentina. PLoS Negl Trop Dis. 2009;3: e363. doi:10.1371/journal.pntd.0000363

11. Moncayo Á, Silveira AC. Current epidemiological trends for Chagas disease in Latin America and future challenges in epidemiology, surveillance and health policy. Mem Inst Oswaldo Cruz. Second Edi. 2009;104: 17–30. doi:10.1590/S0074-02762009000900005

12. Kitron U, Cecere MC, Segura EL, Cohen JE, Gu RE. Sustainable vector control and management of Chagas disease in the Gran Chaco , Argentina. Proc Natl Acad Sci U S A. 2007;104: 16194–16199. doi:https://doi.org/10.1073/pnas.0700863104

13. Abad-Franch F, Vega MC, Rolón MS, Santos WS, de Arias AR. Community participation in Chagas disease vector surveillance: Systematic review. PLoS Negl Trop Dis. 2011;5. doi:10.1371/journal.pntd.0001207

14. Aiello AE, Renson A, Zivich PN. Social Media– and Internet-Based Disease Surveillance for Public Health. Annu Rev Public Health. 2020;41: 101–118. doi:10.1146/annurev-publhealth-040119-094402

15. Pollett S, Althouse BM, Forshey B, Rutherford GW, Jarman RG. Internet-based biosurveillance methods for vector-borne diseases: Are they novel public health tools or just novelties? PLoS Negl Trop Dis. 2017;11: e0005871. doi:10.1371/journal.pntd.0005871

16. Hamer SA, Curtis-Robles R, Hamer GL. Contributions of citizen scientists to arthropod vector data in the age of digital epidemiology. Curr Opin Insect Sci. 2018;28: 98–104. doi:10.1016/j.cois.2018.05.005

17. Fernandez MP, Bron GM, Kache PA, Larson SR, Maus A, Gustafson Jr D, et al. Usability and Feasibility of a Smartphone App to Assess Human Behavioral Factors Associated with Tick Exposure (The Tick App): Quantitative and Qualitative Study. JMIR Mhealth Uhealth. 2019;7: e14769. doi:10.2196/14769

18. Balsalobre A, Ceccarelli S, Cano ME, Ferrari WAO, Cochero J, Martí GA. Apps en el desarrollo de ciencia ciudadana: GeoVin. In: Giacoboni G, editor. I Jornadas de Inclusión de Tecnologías Digitales en la Educación Veterinaria (La Plata, 2018). La Plata: Universidad Nacional de La Plata. Facultad de Ciencias Veterinarias; 2018. pp. 43–44.

19. Wójcik OP, Brownstein JS, Chunara R, Johansson MA. Public health for the people: participatory infectious disease surveillance in the digital age. Emerg Themes Epidemiol. 2014;11: 7. doi:10.1186/1742-7622-11-7

20. Curtis-Robles R, Wozniak EJ, Auckland LD, Hamer GL, Hamer SA. Combining Public Health Education and Disease Ecology Research: Using Citizen Science to Assess Chagas Disease Entomological Risk in Texas. PLoS Negl Trop Dis. 2015;9: e0004235. doi:10.1371/journal.pntd.0004235

21. Palmer JRB, Oltra A, Collantes F, Delgado JA, Lucientes J, Delacour S, et al. Citizen science provides a reliable and scalable tool to track disease-carrying mosquitoes. Nat Commun. 2017;8: 916. doi:10.1038/s41467-017-00914-9

22. Holston J, Suazo-Laguna H, Harris E, Coloma J. DengueChat: A Social and Software Platform for Community-based Arbovirus Vector Control. Am J Trop Med Hyg. 2021;105: 1521–1535. doi:10.4269/ajtmh.20-0808

23. Delgado-Noguera LA, Hernández-Pereira CE, Ramírez JD, Hernández C, Velasquez-Ortíz N, Clavijo J, et al. Tele-entomology and tele-parasitology: A citizen science-based approach for surveillance and control of Chagas disease in Venezuela. Parasite Epidemiol Control. 2022;19: e00273. doi:10.1016/j.parepi.2022.e00273

24. Levy M, Paz-Soldan VA. An immune system for the city: a new paradigm for control of urban disease vectors. 2023.

25. Instituto Nacional de Estadística e Informatica (INEI). Arequipa resultados definitivos Tomo I. Lima; 2018 Oct.

26. Barbu CM, Buttenheim AM, Pumahuanca M-LH, Calderón JEQ, Salazar R, Carrión M, et al. Residual Infestation and Recolonization during Urban *Triatoma infestans* Bug Control Campaign, Peru1. Emerg Infect Dis. 2014;20: 2055–2063. doi:10.3201/eid2012.131820

27. Department of Health and Mental Hygiene of New York City. Cómo prevenir y eliminar los chinches de manera segura. New York: Department of Health and Mental Hygiene of New York City; 2014.

28. Gurtler RE, Cohen JE, Cecere MC, Lauricella MA, Chuit R, Segura EL. Influence of humans and domestic animals on the household prevalence of Trypanosoma cruzi in Triatoma infestans populations in northwest Argentina. American Journal of Tropical Medicine and Hygiene. 1998;58: 748–758. doi:10.4269/ajtmh.1998.58.748

29. Michie S, van Stralen MM, West R. The behaviour change wheel: A new method for characterising and designing behaviour change interventions. Implementation Science. 2011;6: 42. doi:10.1186/1748-5908-6-42

30. Gilmore B, Ndejjo R, Tchetchia A, de Claro V, Mago E, Diallo AA, et al. Community engagement for COVID-19 prevention and control: a rapid evidence synthesis. BMJ Glob Health. 2020;5: e003188. doi:10.1136/bmjgh-2020-003188

31. Sousa LB, Craig A, Chitkara U, Fricker S, Webb C, Williams C, et al. Methodological Diversity in Citizen Science Mosquito Surveillance: A Scoping Review. Citiz Sci. 2022;7: 8. doi:10.5334/cstp.469

32. El peruano. Facebook es la red social más popular en el Perú. El peruano. 16 Mar 2020.

33. El comercio. Osiptel: El 88,4% de los hogares peruanos cuenta con un smartphone. El comercio. 19 Jul 2022.
